# Boosting plant food polyphenol concentration by saline eustress as supplement strategies for the prevention of metabolic syndrome: an example of randomized interventional trial in the adult population

**DOI:** 10.3389/fnut.2023.1288064

**Published:** 2023-12-22

**Authors:** Vincenzo Ferrantelli, Sonya Vasto, Angelina Alongi, Leo Sabatino, Davide Baldassano, Rosalia Caldarella, Rosaria Gagliano, Luigi Di Rosa, Beppe Benedetto Consentino, Lorena Vultaggio, Sara Baldassano

**Affiliations:** ^1^Experimental Zooprophylactic Institute of Sicily, Palermo, Italy; ^2^Euro-Mediterranean Institutes of Science and Technology (IEMEST), Palermo, Italy; ^3^Department of Biological, Chemical and Pharmaceutical Sciences and Technologies, University of Palermo, Palermo, Italy; ^4^Dipartimento Scienze Agrarie, Alimentari e Forestali, University of Palermo, Palermo, Italy; ^5^Department of Promoting Health, Maternal-Infant, Excellence and Internal and Specialized Medicine (ProMISE) G. D'Alessandro, University of Palermo, Palermo, Italy; ^6^Department of Laboratory Medicine, “P. Giaccone” University Hospital, Palermo, Italy

**Keywords:** MetS, diet, body homeostasis, functional food, lettuce, phytochemicals, nutritional intervention

## Abstract

**Introduction:**

Phenolic compounds in lettuce can increase by the application of positive stress (eustress) such as moderate saline stress. Phenolic compounds possess antioxidant capacity that is a key factor in the detoxification of excess reactive oxygen species. A double-blinded randomized interventional and placebo- controlled study design was carried out to compare the effect of daily dietary eustress lettuce ingestion in hepatic, lipid, bone, glucose, and iron metabolism.

**Methods:**

Forty-two healthy volunteers, 19 female and 23 male participants, were divided into two groups. Participants were randomized into a polyphenol-enriched treatment (PET) arm or control arm. Each arm consumed 100 g/day of control or eustress (polyphenols enriched treatment = PET) lettuce for 12 days. Primary study outcomes were serological analysis for assessing hepatic, lipid, bone, iron, and glucose markers at baseline and after 12 days. Secondary outcomes assessed body composition.

**Results:**

Salinity stress reduced plant yield but increased caffeic acid (+467%), chlorogenic acid (+320%), quercetin (+538%), and rutin (+1,095%) concentrations. The intake of PET lettuce reduced PTH, low-density lipoprotein (LDL), cholesterol, alanine transaminase (ALT), and aspartate transaminase (AST) enzyme levels and increased vitamin D and phosphate levels, while iron and glucose metabolism were unaffected.

**Discussion:**

Supplementation with eustress lettuce by increasing polyphenols concentration ameliorates hepatic, lipid, and bone homeostasis. Body composition was not affected.

**Clinical trial registration:**

https://classic.clinicaltrials.gov/ct2/show/NCT06002672, identifier: NCT06002672.

## 1 Introduction

In recent years, the demand for fresh vegetables has been increasing due to consumers' self-awareness that fibers, mineral salts, and vitamins have a beneficial effect on health by reducing, for example, the risk of age-related diseases (1). Due to their bioactive properties, horticultural crops are in the interest of the major lines of international food manufacturing. Therefore, farmers together with scientists are working to characterize new market products with enhanced nutraceutical activity (2) in order to maintain sustainability for the preservation of natural resources.

In this view, it is necessary to point out the gap between research and market offering (3). At present, horticultural crops are well characterized for the quality component, while the information about the functional properties comes from *in vitro* (cells) or *in vivo* animal studies (rodents) and, therefore, translated to human metabolism. Thus, the effects of crops enriched with nutraceutical compounds on human body homeostasis and on disease prevention need to be addressed.

Lettuce (*Lactuca sativa* L.) is a green leafy vegetable belonging to the *Asteraceae* family, which is consumed worldwide by most of the population in all seasons. The high fiber content, which promotes satiety, and the presence of vitamin C, flavonoids, and phenolic acids place lettuce at the top of healthy food items (4, 5).

Groundwater salinization caused by salts such as NaCl and CaCl^2^ is a growing concern in several regions of the ecosphere, comprising those of the Mediterranean basin (6). These salts can be derived from different fonts such as seawater infiltration, dissolution of minerals in rocks, and anthropogenic actions (7). The high electrical conductivity of irrigation water and agricultural soils is mainly due to the excessive use of fertilizers and determines salt stress in plants, affecting their growth and yield. However, the salinity can also be used to increase the content of bioactive compounds (8). The application of positive stress (eustress) can modulate the biosynthesis and accumulation of secondary metabolites *via* the activation of plant defense mechanisms (9). Therefore, the nutritional management of horticultural crops and the application of eustress, such as salinity, provide valuable and cost-effective tools to manipulate plant phytochemical content and product quality, which will contribute to meeting the growing market trends toward high value-added products (8). In fact, it was shown that moderate salinity resulted in an increase of ascorbic acid, α-tocopherol content, and antioxidant activity of *Cichorium spinosum* leaves (10). Furthermore, an increase in anthocyanin and ascorbic acid contents in green and red lettuce leaves (11, 12) was documented.

Several studies strongly suggest that a diet rich in polyphenols may have beneficial effects in the prevention of metabolic syndrome and related diseases (13), including cancer. Data on the effects of the consumption of polyphenols-enriched lettuce on human health are missing. Therefore, the specific objective of this study was to test whether polyphenols-enriched treatment (PET) lettuce supplementation influences human health. Two outcomes were measured: Primary outcomes investigating whether the consumption of PET lettuce could influence key regulators of body homeostasis, in particular, liver, lipid, bone, glucose, and iron metabolism in the adult population by assessing serological levels of hepatic, lipid, bone, iron, and glucose markers at baseline and after 12 days. Secondary outcomes evaluated the body composition.

## 2 Materials and methods

### 2.1 Cultivation practice, experimental site, and design and yield of lettuce

The lettuce plants were grown on a farm belonging to the Department of Agricultural, Food, and Forestry Sciences of the University of Palermo (SAAF), located near Palermo. The growing cycle was carried out in a tunnel covered with a transparent polyethylene film. Before transplanting, aged manure was added as a soil amendment at a rate of 10 t ha^−1^. On March 15, 2021, 300 plug plants of “Canasta” lettuce (*Lactuca sativa* L.) (Syngenta Seed, Basel, Switzerland), at the stage of four to five true leaves, were transplanted at a density of 20 plants m^−2^. Considering the short duration of the lettuce growing cycle and the nitrogen available in the soil (2‰), no fertilizers were added. During the growing cycle, the irrigation management was conducted in accordance with standard lettuce cultivation practices. After 7 days of transplanting, half of the plants were irrigated with tap water (0 mM NaCl; EC 0.68 dS m^−1^), while the remaining plants were exposed to salinity stress (25 mM NaCl; EC 3.15 dS m^−1^). All plants were harvested on May 31, 2021 and immediately weighed (10 plants per replication) and transferred to the Department of Biological, Chemical, and Pharmaceutical Sciences and Technologies, University of Palermo to start the clinical trial. The agronomic trial was arranged in a randomized complete block design, and each block was replicated three times, containing 50 plants each.

### 2.2 Design of the study and participants

This is a randomized and placebo-controlled study design. The protocol was approved with the number of approbation protocol 02/2020 by the Ethics Committee of the University of Palermo Hospital P. Giaccone and was conducted in accordance with the Declaration of Helsinki. Participants provided, prior to study inclusion, written informed consent. The participants were recruited in March 2021. The study is registered at clinicaltrial.gov NCT06002672.

**Figure 1:**
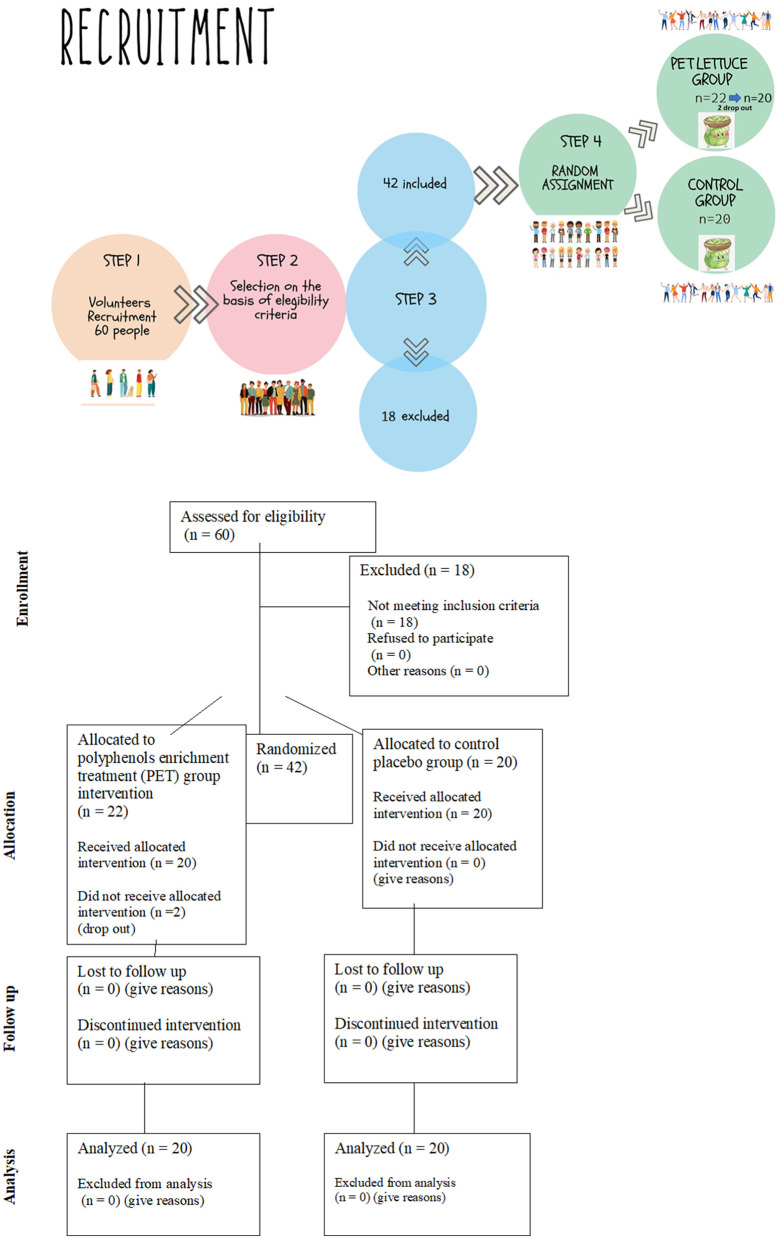
Flow chart recruitment and random assignment of participants to the study.

Based on previous studies (14–16), for the estimation of fasting insulin levels, *a priori* power calculation utilizing a level of statistical significance α of 5% and a probability β of 20% was performed, which estimated a sample size of eight people. In order to reduce type two error risks and to improve the evaluation power for the secondary outcomes, we decided to include not <12 persons in each group of the study. Forty-two healthy volunteers, 19 female and 23 male participants, joined the study. A team of physicians and nutritionists individually instructed the volunteers for the compilation of a food and lifestyle diary and for not changing lifestyle habits throughout the study period. This is because having a trained interviewer review increases the quality of the report (17). The information about food diaries was collected 8 days before the beginning of the study as previously reported (15) and until the end of the study in order to monitor compliance and adherence, showing eventual food habits and lifestyle modification. More specifically, participants were asked to record in detail the foods and beverages they consumed during the day at designated times and to report physical activity type and duration. The team of nutritionists conducted meal plan analysis of food diaries for each participant to monitor energy, macronutrients, micronutrients, and water and fiber consumption by using the WinFood software from Medimatica s.r.l Colonnella (TE) (Supplementary Table 1). This was done in order to check that subjects maintained their usual diet and lifestyle during the supplementation period. The flowchart of the recruitment and assignment of applicants to study groups is described in Figure 1. The criteria of eligibility for participation in the study were the absence of gastrointestinal, cardiac and blood dysfunction, absence of metabolic disorders, viral infection, use of medication including minerals and vitamins supplements and exogenous hormones, and the absence of pregnancy and breastfeeding condition. The criteria are summarized in Table 1.

**Table 1:** Criteria of eligibility for participation in the recruitment process.

**Criteria of selection for participation in the study**	**Inclusion criteria**	**Exclusion criteria**
Italian ethnicity	Absence of gastrointestinal, cardiac and blood dysfunction, absence of metabolic disorders, and viral infection	Presence of metabolic or chronic disease
Not taking medications and supplements	Age range: 18–65 years	Use of vitamins and minerals supplements and use of medications
Clinically healthy	Body mass index: 18.5–28 kg/m^2^	Pregnancy and breastfeeding condition

### 2.3 Experimental research design procedures

**Figure 2:**
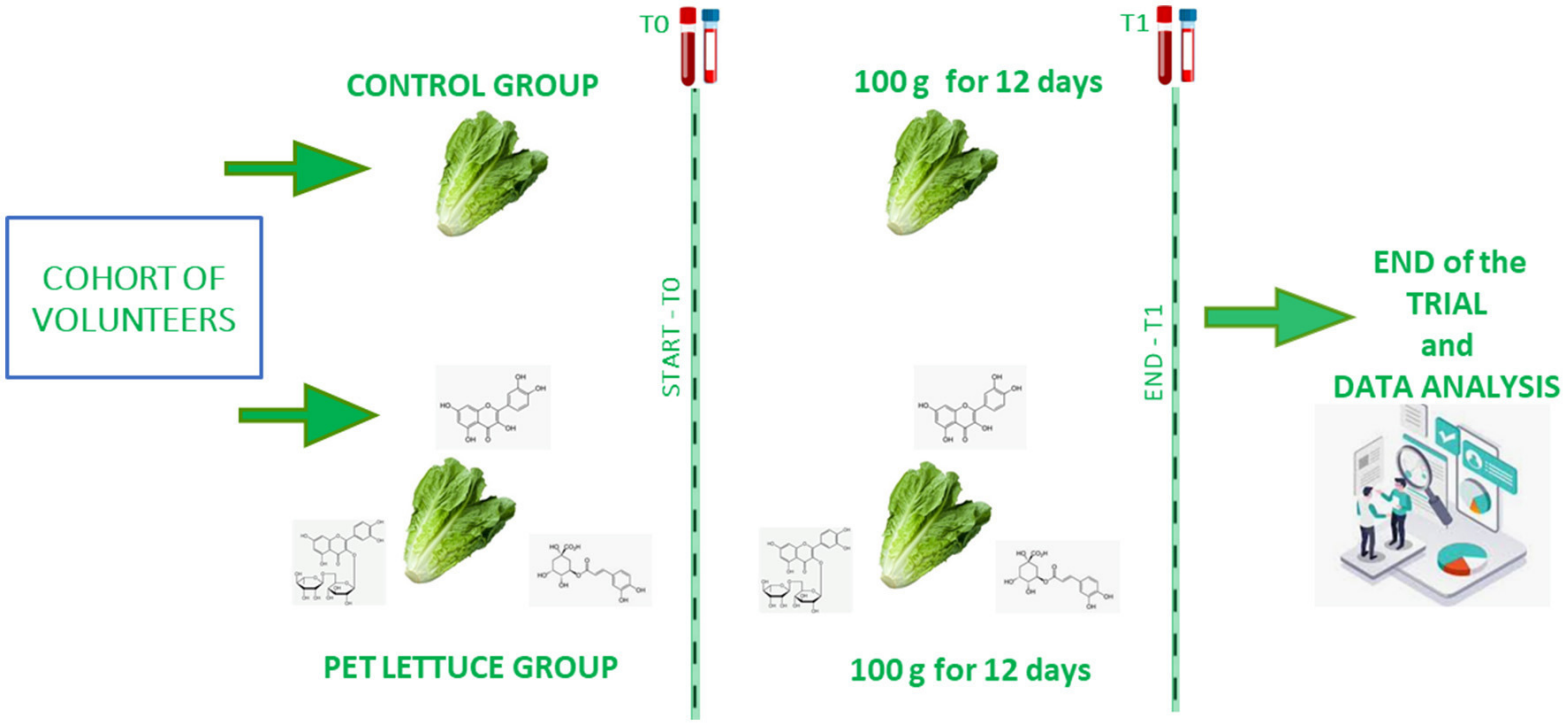
Schematic overview of the nutritional interventional in the control and eustress polyphenols-enriched treatment (PET) lettuce groups of study. Canasta lettuce (above) or PET lettuce (below) was administered to the volunteers every day, 100 g a day, for a total of 12 days. The samples of blood were collected at time zero (start or baseline) and at time one (the end of the intervention).

Participants attended the ambulatory Nutrition Age and Bone (NABbio) of the University of Palermo, STEBICEF department in the morning between 7:00 and 8:00 a.m. (18, 19) under controlled conditions. They were in fasting condition from the dinner of the day before (12-h fasting) as previously reported (15). A sample of venous whole blood was collected in the appropriate tubes for serum and plasma, and the anthropometric measurements including body lean and fat mass, weight, and height (20) were recorded for each participant. To randomize the participants, a random number generator computer program (excel) was used to generate random numbers from the list for each group. Then, the participants were randomly assigned to the control or experimental group and given random numbers by a third party, who encoded the lettuces with matching random numbers. The medical staff, the investigator, and the participants in the study were blinded to the allocation during the whole data collection period. The investigators were also blinded during the data analysis and the sample assessment. Thus, participants in a double-blinded manner were allocated to one group, were provided with the crops of lettuce (~2 kg), were instructed to store them (21), and to eat 100 g each day for 12 days. In the control group, 12 volunteers (8 female and 12 male participants) who received canasta lettuce were allocated. In the PET lettuce group, 22 people (11 female and 11 male participants) were allocated, but 2 subjects dropped out due to personal problems, thus leaving a total of 20 volunteers (9 female and 11 male participants). The study outcome is represented by a sample of blood collected at time zero and after 12 days of nutritional intervention (15), as shown in Figure 2. Specifically, the primary outcome assessment involved measuring the serological levels of hepatic, lipid, bone, iron, and glucose markers at baseline and after 12 days. secondary outcome measured body mass.

Authors had no access to information that could identify individual participants during or after data collection. To not be individuated and be anonymous, the samples were identified by code numbers.

### 2.4 Hematological and biochemical analysis of the samples

For the analysis of the samples, to obtain serum, blood was collected in specific tubes without anti-coagulant and centrifugated at room temperature, 1.300 g for 15 min. An automated procedure on the Roche COBAS c503, according to standard commercially available assays supplied by Roche Diagnostics, was performed to measure glucose and insulin, markers of hepatic, lipid, and iron metabolism, and markers of bone metabolism (14–16, 20, 22, 23).

### 2.5 Analysis of polyphenols in lettuces

Lettuce samples were stored immediately on reception at −80°C in a properly numbered plastic jar until analysis. Samples were properly homogenized before analysis using a laboratory blender. The blank was analyzed to confirm the absence of polyphenols.

In the sample extraction procedure, 0.1 g of lettuce grounded sample was used. Then, 10 mL of acetone/water/HCl (70:29.9:0.1 v/v/v) solution was added, and this mixture was shaken in a vortex, sonicated for 30 min, and centrifuged at 3,500 rpm for 15 min at +5°C. The supernatant was incubated overnight at 4°C. After incubation, samples were filtered using a 0.45 μ filter, and finally, 10 μL of the extract was injected into the UHPLC/HRMS system. For the analysis of polyphenols in lettuces, an accela pump HPLC binary pump, with an XBridge BEH column (Thermo) C18 2.5 μm (2.1 × 50 mm) was used. The oven temperature was +40°C and the flow rate was 300 μL min^−1^. The mobile phases were aqueous 0.1% formic acid (A) and ACN hypergrade (B), and the gradient program was as follows (time min, % B): (0, 5); (0.33, 30); (1.63, 100); (9.63, 100); (11.63, 5). The total run time was 13.63 min. The chromatographic system was coupled to a hybrid quadrupole-Orbitrap HRMS instrument (Q-Exactive, Thermo Scientific, Bremen, Germany), with heated electrospray ionization (HESI) in the positive and negative ionization mode. The full MS-ddMS2 acquisition mode was applied with a resolution of 70,000 (m/z 200, FWHM), automatic gain control (AGC) target 3e6, maximum injection time (IT) 200 ms, and a scan range of 100–1,000 m/z. Nitrogen was obtained from a Zephyr generator (Thermo Fisher Scientific, San Jose, CA, USA). Compounds were identified by retention time (RT) and accurate mass. In the experiment, a mass accuracy of 5 ppm was used. The analysis was carried out on the basis of calibration curves (https://doi.org/10.6084/m9.figshare.24219346).

### 2.6 Statistical analyses

Student *t*-tests were used for the comparison of polyphenols in control and eustress lettuce. For the human study, the groups of study were first compared at baseline by using Student *t*-tests, while the differences between T0 and 1 were compared by using one-way ANOVA followed by Tukey's posttest by using GraphPad Prism software. A *p*-value for a dataset of ≤ 0.05 indicates that the results were statistically significant. Data are expressed as mean ± standard deviation (S.D).

## 3 Results

### 3.1 Phenolic quantification analysis in lettuces

The quantification of the phenolic compounds was performed in control and eustress (polyphenols enriched treatment) lettuces in order to observe differences in the concentration of polyphenols. Eustress (polyphenols enriched treatment) lettuce showed significantly increased concentrations of caffeic acid, chlorogenic acid, quercetin, and rutin compared to the control lettuce. No difference was observed in apigenin and catechin concentrations between eustress lettuce and control (Table 2).

**Table 2:** Polyphenols quantification in control and eustress (polyphenols enriched treatment PET) lettuce.

**Polyphenols content (μg/100 g of fresh weight)**	**Control lettuce mean ±SEM**	**PET lettuce mean ±SEM**	***p*-value**
Apigenin	114.4 ± 4.0	107.4 ± 1.2	n.s.
Caffeic acid	27.4 ± 1.1	128 ± 0.8	<0.000001
Catechin	18 ± 4.2	25.1 ± 2.0	n.s.
Chlorogenic acid	260 ± 4.1	834.1 ± 96.6	0.000144
Quercetin	28.2 ± 3.3	151.8 ± 7.8	<0.000001
Rutin	14.6 ± 1.6	160 ± 5.9	<0.000001
Total polyphenols	462.4 ± 7.0	1406.5 ± 106.9	0.000005

Data are means ± SEM. Student t-tests were used to compare the two groups of study. A p-value higher than 0.05 denotes that the change is not statistically significant (n.s.) and indicates strong evidence for the null hypothesis.

### 3.2 Anthropometric characteristics of the study population

As shown in Table 3, the two groups of the study were homogeneous in nature. The study started in March 2021 and ended in June 2021. No difference was observed in the percent of body fat and lean mass, in body weight among the two groups of subjects enrolled in the study at baseline and at the end of the intervention (Table 3).

**Table 3:** Characteristics of subjects in the two groups (control and polyphenols-enriched treatment, PET lettuce groups) at baseline (T0) and following 12 days of lettuce administration (100 g/day) (T1).

**Study group information**	**Control group T0 (*n* = 20; 8 females; 12 males) Mean ±S.D**.	**PET lettuce group T0 (*n* = 20; 9 females; 11 males) Mean ±S.D**.	**Control group T1 (*n* = 20; 8 females; 12 males) Mean ±S.D**.	**PET lettuce group T1 (*n* = 20; 9 females; 11 males) Mean ±S.D**.	***p*-value**
Age (years)	45 ± 12	37 ± 11	-	-	n.s.
Body weight (kilograms)	75 ± 13	72 ± 15	75 ± 12	72 ± 15	n.s.
Body mass index	28.2 ± 3.8	26.0 ± 5.1	-	-	n.s.
Height (centimeters)	164 ± 8	166 ± 8	-	-	n.s.
Body fat mass (percent)	24.2 ± 4.7	23.4 ± 4.2	24.3 ± 4.8	23.6 ± 4.3	n.s.
Body lean mass (percent)	75.7 ± 4.7	76.5 ± 4.2	75.7 ± 4.8	76.4 ± 4.3	n.s.

All the values are indicated as means ± standard deviations (SD). Student t-tests were used to compare control and PET lettuce groups at baseline. When appropriate differences between and within the groups (T0 and T1) were compared by using one-way ANOVA followed by Tukey's posttest. A p-value higher than 0.05 means that the change is not statistically significant and reported as not statistically significant (n.s.). A p-value for a dataset of ≤ 0.05 indicates that the results were statistically significant.

### 3.3 PET lettuce consumption in adults and its impact on hepatic and lipid homeostasis

PET-enriched lettuce consumption, 100 g/day, for 12 days improved liver function. Specifically, a reduction of ~30% in AST and ~34% in ALT enzymatic levels following 12 days of PET lettuce consumption from baseline (Table 4) was observed. Ingestion for 12 days of control lettuce was without effects on hepatic function. No difference was observed in ALP, GGT, TP, and albumin within or among the two groups (Table 4). The nutritional intervention with PET lettuce ameliorates lipid metabolism. In fact, it reduced total cholesterol and LDL levels and did not affect triglycerides and HDL concentration (Table 5).

**Table 4:** Markers of hepatic function were measured in the study population baseline (T0) and following 12 days of control and polyphenols-enriched treatment (PET) lettuce administration (100 g/day) (T1).

**Markers of hepatic function**	**Control group T0 (*n* = 20) Mean ±SD**	**Control group T1 (*n* = 20) Mean ±SD**	***p*-value**	**PET lettuce group T0 (*n* = 20) Mean ±SD**	**PET lettuce group T1 (*n* = 20) Mean ±SD**	***p*-value**
Aspartate aminotransferase (AST) (U/l)	24 ± 7	25.6 ± 6	n.s.	23 ± 7	16.4 ± 5	**0.0108**
Alanine aminotransferase (ALT) (U/l)	25 ± 7.0	23.6 ± 8.0	n.s.	24 ± 8.4	15.8 ± 11	**0.0309**
Alkaline phosphatase (ALP) (U/l)	66 ± 9	61 ± 11	n.s.	66 ± 11	64 ± 13	n.s.
Gamma-glutamyl transferase (GGT) (U/L)	17 ± 6	17.5 ± 6	n.s.	17 ± 10	15 ± 7	n.s.
Total protein (TP) (g/L)	72 ± 5	71 ± 4	n.s.	72 ± 3	70 ± 3	n.s.
Albumin (g/L)	46 ± 3	44 ± 3	n.s.	45 ± 2.4	44 ± 2.5	n.s.

All the values are indicated as means ± standard deviations (SD). Differences between and within the groups (T0 and T1) were compared by using one-way ANOVA followed by Tukey's posttest. A p-value higher than 0.05 means that the change is not statistically significant and reported as not statistically significant (n.s.). A p-value for dataset ≤ 0.05 indicates that the results were statistically significant.

**Table 5:** Markers of lipid metabolism were measured in the study population baseline (T0) and following 12 days of control and polyphenols-enriched treatment (PET) lettuce administration (100 g/day) (T1).

**Markers of lipid metabolism**	**Control group T0 (*n* = 20) Mean ±SD**	**Control group T1 (*n* = 20) Mean ±SD**	***p*-value**	**PET lettuce group T0 (*n* = 20) Mean ±SD**	**PET lettuce group T1 (*n* = 20) Mean ±SD**	***p*-value**
Triglycerides (mg/dL)	104 ± 49	97 ± 20	n.s.	91 ± 56	95 ± 29	n.s.
Total cholesterol (mg/dL)	194 ± 27	189 ± 14	n.s.	198 ± 32	167 ± 33	0.0035
Low-density lipoproteins (LDL) cholesterol (mg/dL)	116 ± 28	113 ± 24	n.s.	124 ± 32	90 ± 27	0.0010
High-density lipoproteins (HDL) cholesterol (mg/dL)	65 ± 13	61 ± 13	n.s.	63 ± 11	60 ± 14	n.s.

All the values are indicated as means ± standard deviations (SD). Differences between and within the groups (T0 and T1) were compared by using one-way ANOVA followed by Tukey's posttest. A p-value higher than 0.05 means that the change is not statistically significant and reported as not statistically significant (n.s.). A p-value for dataset ≤ 0.05 indicates that the results were statistically significant.

### 3.4 PET lettuce intake in the adult population and its impact on bone homeostasis

Daily intake of PET lettuces reduced serum PTH and increased vitamin D and phosphate levels compared to baseline and control lettuce (both baseline and after 12 days) (Table 6). However, there was no modification in the markers of bone remodeling. There was no change in serum levels of bone formation (osteocalcin) and resorption (CTX), calcium, potassium, and calcitonin compared to the baseline or control group.

**Table 6:** Markers of bone remodeling and metabolism were measured in the study population baseline (T0) and following 12 days of control and polyphenols-enriched treatment (PET) lettuce administration (100 g/day) (T1).

**Markers of bone metabolism**	**Control group T0 (*n* = 20) Mean ±SD**	**Control group T1 (*n* = 20) Mean ±SD**	***p*-value**	**PET lettuce group T0 (*n* = 20) Mean ±SD**	**PET lettuce group T1 (*n* = 20) Mean ±SD**	***p*-value**
Carboxy-terminal collagen crosslinks (CTX) (μg/L)	0.45 ± 0.1	0.43 ± 0.1	n.s.	0.5 ± 0.2	0.46 ± 0.2	n.s.
Osteocalcin (μg/L)	22 ± 6.5	20.5 ± 6.2	n.s.	24 ± 6.6	23.3 ± 8	n.s.
PTH (ng/ml)	43.2 ± 9.3	40 ± 9.5	n.s.	40.3 ± 12	29.8 ± 11	0.0177
Vitamin D (μg/L)	32.2 ± 4.4	31.6 ± 5.5	ns	32.5 ± 3.3	41.3 ± 8.9	<0.0001
Phosphate (mg/dL)	3.2 ± 0.7	3.1 ± 0.8	n.s.	3.2 ± 0.6	3.8 ± 0.5	0.0079
Aa calcium (mg/dL)	8.7 ± 1.3	8.4 ± 1.1	n.s.	9.1 ± 0.2	9.2 ± 0.2	n.s.
Potassium (mmol/L)	4.0 ± 0.3	3.9 ± 0.4	n.s.	3.7 ± 1.1	4.2 ± 0.3	n.s.
Calcitonin (ng/L)	2.3 ± 2.4	1.6 ± 1.5	n.s.	2.3 ± 2.3	1.6 ± 1.4	n.s.

All the values are indicated as means ± standard deviations (SD). Differences between and within the groups (T0 and T1) were compared by using one-way ANOVA followed by Tukey's posttest. A p-value higher than 0.05 means that the change is not statistically significant and reported as not statistically significant (n.s.). A p-value for dataset ≤ 0.05 indicates that the results were statistically significant.

### 3.5 PET lettuce intake in the adult population and impact on glucose and iron homeostasis

PET lettuce intake did not affect blood glucose concentration. In fact, no differences were observed in fasting glucose and insulin levels among the two groups of study that consumed control or PET lettuce (Table 7). Moreover, the nutritional intervention with PET lettuce did not modify markers of iron metabolism such as iron, ferritin, and transferrin serum level concentration as well as transferrin saturation if compared to the control arm (Table 7).

**Table 7:** Markers of glucose and iron metabolism were measured in the study population baseline (T0) and following 12 days of control and polyphenols-enriched treatment (PET) lettuce administration (100 g/day) (T1).

**Markers of glucose and iron metabolism**	**Control group T0 (*n* = 20) Mean ±SD**	**Control group T1 (*n* = 20) Mean ±SD**	***p*-value**	**PET lettuce group T0 (*n* = 20) Mean ±SD**	**PET lettuce group T1 (*n* = 20) Mean ±SD**	***p*-value**
Glucose (mg/dL)	85.7 ± 8.4	86 ± 9.7	n.s.	87.7 ± 9.0	88.6 ± 9.7	n.s.
Insulin (mUI/L)	9.2 ± 3.5	9.4 ± 3	n.s.	8.7 ± 4	8.5 ± 5.7	n.s.
Iron (μg/dL)	80 ± 21	75 ± 17	n.s.	88 ± 43	83 ± 36	n.s.
Ferritin (ng/dL)	87 ± 29	84 ± 19	n.s.	81 ± 38	83 ± 17	n.s.
Transferrin (mg/dL)	250 ± 42	242 ± 37	n.s.	267 ± 39	254 ± 41	n.s.
Transferrin saturation (%)	22 ± 7	19 ± 3.5	n.s.	24 ± 9.5	23 ± 10	n.s.

All the values are indicated as means ± standard deviations (SD). Differences between and within the groups (T0 and T1) were compared by using one-way ANOVA followed by Tukey's posttest. A p-value higher than 0.05 means that the change is not statistically significant and reported as not statistically significant (n.s.). A p-value for dataset ≤ 0.05 indicates that the results were statistically significant.

## 4 Discussion

We are dealing with global warming and new pathology, and the COVID-19 pandemic is the latest example. These are signals that there is a need to increase sustainability. Therefore, new approaches to vegetable growth and novelty in their consumption are necessary in order to find new treatments for the prevention of metabolic syndrome. In the present study, we aimed to understand the effect of natural eustress, such as a mild increase in salinity, in one of the most popular consumed vegetables such as lettuce. In particular, the novelty of the study was to verify the ability of eustress to increase functional content, in particular, polyphenols, and to analyze the impact on human physiological homeostasis in an attempt to find new nutritional strategies for the prevention of metabolic syndrome.

Data showed that salinity-stressed plants had a lower yield than control plants. These findings are coherent with a previous study (24) that reported a yield reduction in iceberg lettuce plants stressed with NaCl. This effect could be attributed to the osmotic, ionic, and oxidative stress caused by salinity (25). Moreover, eustress significantly increased the concentration of caffeic acid, chlorogenic acid, quercetin, and rutin with respect to control lettuce. Therefore, as previously shown by Santander et al. (26), we confirm that mild eustress improves the functional quality of lettuce and specifically it acts by increasing polyphenol concentration. This effect is linked to the modification of plant physiological mechanisms and metabolism, permitting plants to grow better under sub-optimal environmental conditions through the activation of antioxidant systems (8).

To investigate the potential effect of PET lettuce supplementation in the prevention of metabolic syndrome, the cohort of adults consumed two different types of lettuce every day in a quantity that we know is well accepted by the participants (23), 100 g a day for 12 days. One group ate the control lettuce, which was grown without salinity eustress, while the other group consumed—for the same period—the PET lettuce. The two groups of study have a similar age range and anthropometric characteristics. The participants did not report differences in the taste or appearance of lettuces. Moreover, they did not report differences in diet and/or lifestyle during the supplementation periods as reported in the food diary. Both the groups, control and PET lettuce, maintained their usual lifestyle. From the physiopathology point of view, the group that consumed the PET lettuce showed improved body homeostasis. In fact, PET lettuce consumption ameliorated liver function by reducing the AST and ALT levels. It also acted in lipid metabolism by reducing total cholesterol and LDL compared with control lettuce. Our results are in accordance with several reports (27), which showed that polyphenols, due to their beneficial properties when regularly consumed in humans, reduced the risk of several metabolic disorders associated with non-alcoholic fatty liver disease. The added value of this study is that polyphenols are naturally increased by eustress, and lettuce can be easily consumed during meals with a normal diet. Thus, regular PET lettuce consumption could be an optimal solution for the prevention of NAFLD and metabolic syndrome.

Despite the health-promoting qualities of polyphenols, it was postulated that the consumption of polyphenols may be associated with decreasing the absorption and bioavailability of iron (28, 29) and affects iron metabolism. Therefore, the markers of iron metabolism were measured in order to verify if consumption of eustress lettuce may be associated with negative health consequences. We did not find differences in iron, transferrin, ferritin, and ceruloplasmin levels between the groups of study ruling out that PET lettuce consumption could interfere with iron status. We also did not find modification in glucose and insulin circulating levels between the control and PET lettuce group. The effects of polyphenols-enriched food in glycemic control during clinical trials are still unclear (30).

In consideration of the tight link between lipid and bone metabolism (31–33) and the lack of clinical trials to define a clear link between these phytonutrients and bone health (34), we analyzed if consumption of PET lettuce impacts markers of bone remodeling and metabolism. We measured serum PTH. This hormone is secreted from the parathyroid glands and is a key regulator of bone metabolism and calcium–phosphorus homeostasis (35). It upregulates bone turnover (36). In particular, PTH has a stimulatory effect on bone resorption. PET lettuce consumption significantly reduced the concentration of PTH. Therefore, its reduction, within the physiological range, suggests that PET lettuce intake exerts a positive effect on the maintenance of skeletal homeostasis (37). In fact, we did not observe a difference in CTX (the marker of bone resorption) and osteocalcin (the marker of bone formation) after 12 days of intervention.

In order to investigate the potential mechanism of action, we measured vitamin D levels. In fact, PTH synthesis and secretion are regulated by vitamin D. Specifically, vitamin D acts at the parathyroid gland by suppressing the synthesis of PTH by repressing its gene (38). In our study, the consumption of eustress lettuce increased vitamin D levels. Therefore, it is possible to suppose that the consumption of PET lettuce by increasing vitamin D levels suppresses the synthesis of PTH (38), and reduces PTH secretion.

Maintenance of skeletal homeostasis through bone remodeling is a tight coupling process that requires subtle coordination between osteoblasts and osteoclasts (39). Following PET lettuce treatment, an increase in the level of phosphate, within the physiological range, in the adult population was observed, while it was not observed for calcium. This is probably because vitamin D is able to enhance the efficiency of intestinal absorption of calcium up to 30–40% and of phosphate to nearly 80% (40). Therefore, we registered significant changes in the level of phosphate but not calcium concentration. Our results are consistent with previous *in vitro* and *in vivo* animal studies, which showed that bioactive phenolics have beneficial effects on bone health (41).

About the mechanism of action by which the supplementation with PET lettuce exerts its effects, we found that it may act at different levels to modulate homeostasis because multiple targets have been reported for polyphenols. They could affect lipid metabolism by modulating oxidative stress (42) by reducing ROS. In fact, polyphenols are powerful regulators of LDL oxidation (43). This could ameliorate lipid metabolism. Moreover, they could impact liver homeostasis by decreasing liver pro-inflammatory cytokines (27) and this in turn could reduce liver enzymes. Elevated liver enzymes often indicate inflammation or damage to liver cells. Polyphenols can protect bone health through modulation of osteoimmunological action, osteoblastogenesis, and osteoclastogenesis. Thus, polyphenols by inducing a reduction of oxidative stress could modulate bone metabolism due to their antioxidant activity and probably also by reduction of inflammation by modulating pro-inflammatory signaling in the bone (41) as they do in liver and adipose tissue. It is interesting to underline that the second cause of osteoporosis is liver disease. In fact, ~30% of patients affected by chronic liver disease suffer from osteoporosis (44). Thus, the use of natural polyphenols, supplied by the vegetable matrix, could be useful in the prevention of liver disease and its associated osteoporosis.

In the PET lettuce, we found an increase in caffeic acid, chlorogenic acid, quercetin, and rutin concentration. Thus, about the mechanism of action, we found that each of them could take part in the observed effects. In fact, caffeic acid is a potent antioxidant and anti-inflammatory molecule. In obese animal models improves lipid profile, and liver biomarker enzymes by decreasing lipoperoxyl radicals (45). In nutritional intervention studies caffeic acid acts by inhibiting low-density lipoprotein (LDL) oxidation and impacts bone metabolism by reducing oxidative stress on bone cells (46). In particular, caffeic acid in *in vitro* and animal models reduces osteoclastogenesis and bone resorption, as well as osteoblast apoptosis (47).

Chlorogenic acid is able to improve lipid metabolism. In diabetic mice, it acts by enhancing fatty acid oxidation and triglycerides lipolysis. Moreover, it reduces liver triglyceride synthesis and fatty acid transportation, alleviating hepatic inflammatory response and oxidative stress (48). Similar to our results, in healthy male subjects (aged 20–31 years), chlorogenic acid, supplied in coffee, decreased LDL-cholesterol (49). In obese rats, it reduced cholesterol and triacylglycerol concentrations in the liver and plasma (50).

About the effects of quercetin, it was found, in accordance with our results, that supplementation with quercetin, in patients with MetS and related disorders, significantly reduced total cholesterol and LDL cholesterol, but did not affect triglycerides and HDL cholesterol (51). In animal models, quercetin exerts antioxidative properties favoring an increase in osteogenic activities and a decrease in osteoclastogenic activities (52).

Rutin enhances proliferation and ossification markers in bone cells. In fact, *in vitro* studies showed that rutin increases osteocyte and osteoblast-related gene expression and, similar to our study, increases vitamin D levels (53). In animal models, rutin reduces reactive oxygen species by inhibiting inflammatory cytokines (54). In rats, rutin reduces plasma total cholesterol and LDL and exerts hepatoprotective effects that seem to be related to antioxidant activity (55).

There are limitations to the clinical trial. The intervention was short-term. It was performed for 12 days. Although it provides information in acute about the effect of PET consumption, longer trials are necessary. Moreover, it could be interesting to analyze the effects of the PET in a cohort of seniors.

To the best of our knowledge, this is the first interventional study that has investigated the benefits of the consumption of dietary polyphenols, carried by a vegetable matrix, in human bone metabolism. The effects that were observed in the liver, and lipid and bone metabolism could pave the way for future application of this natural resource for prevention of osteoporosis and NAFLD.

## Data availability statement

The original contributions presented in the study are publicly available. This data can be found here: https://doi.org/10.6084/m9.figshare.24219346.v1.

## Ethics statement

The studies involving humans were approved by Ethics Committee of the University of Palermo Hospital P. Giaccone. The studies were conducted in accordance with the local legislation and institutional requirements. The participants provided their written informed consent to participate in this study. The study was conducted in accordance with the Declaration of Helsinki and approved by the Ethics Committee of Palermo University Hospital (No. 2/2020).

## Author contributions

VF: Data curation, Formal analysis, Investigation, Writing – review & editing. SV: Conceptualization, Data curation, Investigation, Project administration, Writing – review & editing. AA: Data curation, Formal analysis, Methodology, Validation, Writing – review & editing. LS: Conceptualization, Data curation, Formal analysis, Investigation, Methodology, Writing – review & editing. DB: Data curation, Formal analysis, Investigation, Writing – review & editing. RC: Software, Visualization, Writing – review & editing. RG: Software, Writing – review & editing. LD: Data curation, Formal analysis, Investigation, Methodology, Software, Writing – review & editing. BC: Data curation, Formal analysis, Investigation, Writing – review & editing. LV: Writing – review & editing. SB: Conceptualization, Investigation, Resources, Supervision, Writing – original draft, Writing – review & editing.
