# Supplementary material for: Boosting plant food polyphenol concentration by saline eustress as supplement strategies for the prevention of metabolic syndrome: an example of randomized interventional trial in the adult population
Source: Front Nutr. 2023 Dec 22;10:1288064. doi: 10.3389/fnut.2023.1288064 (PMC10774224; doi:10.3389/fnut.2023.1288064)
Supplement: Supplementary file 1 [file Table_1.DOCX]

**Table 1.** Dietary assessment of subjects in the two groups (Control and Polyphenols Enriched Treatment, PET lettuce groups) at baseline (T0) and following 12 day of lettuce administration (100 grams/day) (T1). All the values are indicated as means ± standard deviations (SD). Student t tests were used to compare control and PET lettuce groups at baseline. When appropriate differences between and within the groups (T0 and T1) were compared by using one-way ANOVA followed by Tukey’s posttest. A p-value higher than 0.05 means that the change is not statistically significant and reported as not statistically significant (n.s.).

| **Dietary assessment** | **Control group T0**  (n=20; 8 females; 12 males)  mean ± S.D. | **PET lettuce group T0**  (n=20; 9 females; 11 males)  mean ± S.D. | **Control group T1**  (n=20; 8 females; 12 males)  mean ± S.D. | **PET lettuce group T1**  (n=20; 9 females; 11 males)  mean ± S.D. | **p-value** |
| --- | --- | --- | --- | --- | --- |
| **Energy intake (kcal/day)** | 1964 ± 455 | 1825 ± 399 | 2001 ±357 | 1947 ± 387 | n.s. |
| **Protein (g/day)** | 69 ± 17 | 66 ± 11 | 71 ± 22 | 70 ± 19 | n.s. |
| **Carbohydrates (g/day)** | 233 ± 60 | 266 ± 44 | 263 ± 81 | 265 ± 41 | n.s. |
| **Fats (g/day)** | 73 ± 22 | 70 ± 25 | 72 ± 31 | 68± 19 | n.s. |
| **Cholesterol (mg/day)** | 211± 110 | 209± 99 | 207± 194 | 206± 34 | n.s. |
| **Monounsaturated fatty acids (g/day)** | 34 ± 11 | 39 ± 34 | 31 ± 14 | 33 ± 34 | n.s. |
| **Fibers (g/day)** | 18 ± 4 | 28 ± 2 | 17 ± 10 | 22 ± 3 | n.s. |
| **Water (liter)** | 1.8 ± 0.2 | 1.7 ± 0.4 | 1.6 ± 0.4 | 1.7 ± 0.1 | n.s. |
